# Rehabilitation workforce descriptors: a scoping review

**DOI:** 10.1186/s12913-022-08531-z

**Published:** 2022-09-17

**Authors:** Thandi Conradie, Karina Berner, Quinette Louw

**Affiliations:** Department of Health and Rehabilitation Sciences, Faculty of Medicine and Health Sciences, Stellenbosch University, Cape Town, South Africa

**Keywords:** Workforce, Rehabilitation, Supply, Indicators, Descriptors

## Abstract

**Background:**

A comprehensive, accurate description of workforce capacity is important for health service planning, to ensure that health services meet local needs. In many low- to middle-income countries, the government’s service planning ability is barred by the lack of accurate and/or comprehensively-described workforce data. In these low-resource settings, lack of appropriate planning leads to limited or no access to rehabilitation services. Variability in the definitions and scope of rehabilitation professionals further complicates the understanding of rehabilitation services and how it should be planned and delivered. Another challenge to describing the primary rehabilitation workforce capacity, is the lack of standardised and agreed-upon global metrics. These inconsistencies highlight the need for a comprehensive understanding of current practices, which can offer guidance to countries wishing to describe their rehabilitation workforce. This study aimed to scope the range of descriptors and metrics used to describe the rehabilitation workforce and to compare the workforce across countries that used similar descriptors in published reports.

**Methods:**

A scoping review was conducted according to the five-step framework first developed by Arksey and O’Malley. The review included a broad search of literature regarding the rehabilitation workforce and how countries quantify and describe the rehabilitation workforce.

**Results:**

Nineteen studies on rehabilitation workforce capacity were identified. All but one (a cross-sectional study) were database reviews. The main descriptors and indicators used to describe the rehabilitation workforce capacity were profession type, age, gender, distributions between urban/rural, level of care, and private/public sectors, absolute count totals, and population-adjusted ratios.

**Conclusion:**

This scoping review provided an overview of descriptors and indicators used to describe the rehabilitation workforce capacity internationally. The study is a first step towards developing standardised descriptors and metrics to quantify the rehabilitation workforce capacity, that will allow for comparison between different settings.

**Trial registration:**

This scoping review protocol has been registered with the Open Science Framework (http://osf.10/7h6xz).

**Supplementary Information:**

The online version contains supplementary material available at 10.1186/s12913-022-08531-z.

## Introduction

A comprehensive and accurate description of workforce capacity is important for health service planning to ensure that health services meet local needs [1]. Health service planning strongly hinges on the appropriate level of human resources to deliver the required health services. In many low- to middle-income countries (LMICs), the government’s ability to plan services is barred by the lack of accurate and/or comprehensively-described workforce data [2]. Although many services are sub-optimal in countries with constrained resources, services such as rehabilitation are particularly affected as it is often under-recognised and not prioritised in countries with limited resources for healthcare delivery [1]. In these low-resource settings, the lack of appropriate planning leads to limited or no access to rehabilitation services.

The limited access to rehabilitation services in LMICs is widely reported in the literature [1, 3], despite the increasing need for rehabilitation services in these settings. Of further concern is that the need for rehabilitation services is increasing at a higher rate in LMICs compared to high-income countries (HICs) [4, 5]. Therefore, ministries of health must understand the rehabilitation workforce, as it should be a fundamental component of health service planning. The importance of describing the rehabilitation workforce is also accentuated by the World Health Organisation’s (WHO) Rehabilitation 2030 initiative, which aims to strengthen rehabilitation in the health system [1]. However, strengthening of rehabilitation in the health system remains a challenge due to limited rehabilitation-related data. Rehabilitation is a complex construct, which remains poorly understood by policymakers. The variability in the definitions and scope of rehabilitation professionals further complicates the understanding of rehabilitation services and how it should be planned and delivered [5–7].

Definitions of the rehabilitation workforce vary across countries due to differences in the classification of rehabilitation professionals [5, 7, 8]. The WHO Rehabilitation Competency Framework highlights the diversity in the rehabilitation workforce between WHO regions [9]. WHO defines a rehabilitation worker as ‘a person delivering or supporting the delivery of rehabilitation, whether interacting directly or indirectly with a person, their family or service-user groups’ [3]. In many countries, rehabilitation professionals are also referred to as ‘allied health workers’ and could also encompass different professionals such as radiographers [8, 10]. The WHO classification of a ‘rehabilitation worker’ also includes other professionals such as nurses and psychologists who specialise in rehabilitation [3, 9]. However, the core professionals classified as rehabilitation professionals in LMICs include occupational therapists, audiologists, speech-language therapists and physiotherapists [3, 9].

Describing the primary rehabilitation workforce capacity is also challenged by a lack of standardised and agreed-upon global metrics [5, 7]. The knowledge base describing the rehabilitation workforce is arguably limited (and biased towards HICs), as there is a paucity of knowledge on workforce metrics to describe the local context, plan services and enable between-country comparisons [5, 7, 8]. Although some descriptors used to describe the rehabilitation workforce are similar in published reports, differences are also apparent [5, 7, 8, 11]. These inconsistencies highlight the need for a comprehensive understanding of current practices, which can offer guidance to countries wishing to describe their rehabilitation workforce [1, 3]. A synthesis of rehabilitation workforce descriptors will be particularly useful to LMICs, to enable the efficient selection of metrics that will suit their local contexts. In addition, it will allow countries to use rehabilitation workforce descriptors that can be used for global comparison and benchmarking [2, 5, 9].

To respond to the WHO’s recommendation for a country-specific description of the rehabilitation workforce, the selection of appropriate and commonly used metrics is an important preliminary step towards service planning [12]. Therefore, this study aimed to scope the range of descriptors and metrics used to describe the rehabilitation workforce in published reports. A secondary objective was to compare the workforce across countries that used similar descriptors in the published reports.

## Methods

### Study design

A scoping review was conducted according to the five-step framework first developed by Arksey and O’Malley [13]. These steps are i) identifying the research objective(s), ii) identifying relevant studies, iii) selecting the studies, iv) charting the data, and v) collating, summarising and reporting the results. The review included a broad search of literature regarding the rehabilitation workforce and how countries quantify and describe the rehabilitation workforce. Reporting followed the PRISMA Extension for Scoping Reviews checklist (see Addendum 1) [14].

### Identifying the research question

The research objectives assisted in determining the eligibility criteria, guided the scope of the review, and determined an effective search strategy.

To achieve the overall study aim, the following objectives were completed:

1. Understand the data collection methods or data sources used to collate rehabilitation workforce capacity information (descriptors and indicators),
2. Synthesise which descriptors and indicators were used to describe (or quantify) national or regional rehabilitation workforce data (e.g., type of therapist, qualifications and work setting).

### Identifying relevant studies

#### Search strategy

A broad and comprehensive three-step search strategy was undertaken to identify published studies in five databases (PubMed, CINAHL, Scopus, Science Direct and Web of Science). These databases were chosen in consultation with a librarian as they yielded the most relevant results. Databases such as Africa Wide and EBSCOHost were accordingly excluded. As a first step, an initial limited keyword search was conducted in PubMed, using key terms related to ‘rehabilitation workforce’, ‘physiotherapy’, ‘physical therapy’, ‘occupational therapy’, ‘speech therapy’, ‘speech-language therapy’, ‘audiology’, ‘allied health’, ‘rehabilitation workforce’, ‘human resources’, ‘human resources for health’, ‘staffing’, ‘supply’, ‘population needs’ and ‘demographics’.

This was followed by an analysis of the text words of the titles and abstracts of potentially relevant articles, and keywords used to index articles. A second search was undertaken, which incorporated the identified additional or refined index and keywords across all databases. The preliminary search string that was developed for PubMed is provided in Addendum 2. Thirdly, all the reference lists of identified literature were searched to include additional evidence that might have been missed in the electronic database searches. A librarian was consulted to ensure that these searches were methodical and transparent. The librarian conducted the searches independently to ensure that the same results were produced as those that the reviewer conducting searches (TC) found.

All database search outcomes were transferred to Rayyan V0.1.0 software (Rayyan Systems Inc., MA, USA) [15]. Deduplication of all articles was conducted using Rayyan before the screening of titles and abstracts.

#### Inclusion and exclusion criteria

##### Type of evidence sources

The scoping review included peer-reviewed primary studies, published in English, on rehabilitation workforce. We considered descriptive, cross-sectional and cohort designs. Grey literature and studies published before 2000 were excluded. Studies where the full texts were not available, were also excluded. No geographical limitations were applied.

##### Types of outcomes

We considered rehabilitation workforce capacity demographics (e.g., profession type, gender, age, employment in the private versus public sector, and employment in a full- versus part-time capacity), and rehabilitation workforce capacity indicators (e.g., the total number of rehabilitation workforce, the ratio per population, rural versus urban distribution or distribution between levels of care). Studies only reporting on forecasting were excluded.

##### Population

Only literature that included the rehabilitation workforce, or at least one of the professions that are classified as rehabilitation professionals in the relevant countries (such as physiotherapy, occupational therapy, speech-language and hearing therapy/pathology and audiology), were considered [16].

#### Study selection

Following deduplication, one reviewer (TC) screened all titles and abstracts retrieved and determined eligibility for inclusion. Eligible titles and abstracts were included for full-text review, which was again screened against the review eligibility criteria. If the reviewer (TC) was unsure of a study’s eligibility, a discussion with the second (QL) and third (KB) reviewers ensued for consensus. Reasons for excluding ineligible studies were discussed with the second (QL) and third (KB) reviewers and were documented.

### Data charting

Based on a discussion between the reviewers (TC, QL and KB), a draft data extraction form was developed in Microsoft Excel to ensure that all relevant data about the study objectives were included in the form. The form was piloted by two reviewers (TC and QL) using five randomly-selected eligible articles. Amendments were made by incorporating additional unforeseen data arising from the articles that were deemed important in terms of contributing to the review objectives. The data from the final sample of included articles were extracted by one reviewer (TC), using the final version of the data extraction form (Addendum 3).

### Data analysis

Descriptive information (including number of included studies, study designs, publication years, study countries, and professional type) was summarised quantitively. Information on data collection methods and data sources that were used to collate the rehabilitation workforce capacity information were described narratively. Demographic and work setting-related descriptions (e.g., urban, rural, private, public, age and gender) of the rehabilitation workforce data were described similarly. Metrics used to describe/quantify the national or regional rehabilitation workforce data were described and synthesised. To enable comparison and ease of interpretation of the findings regarding the workforce supply, percentages were calculated and, where data were available, ratios per 10 000 of the population were determined. Where ratios were reported per 1 000 or 100 000, the ratio was adjusted to 10 000 population. In cases where studies conducted retrospective analyses, the most recent year’s data were reported.

## Results

### Selection of studies

**Fig. 1:**
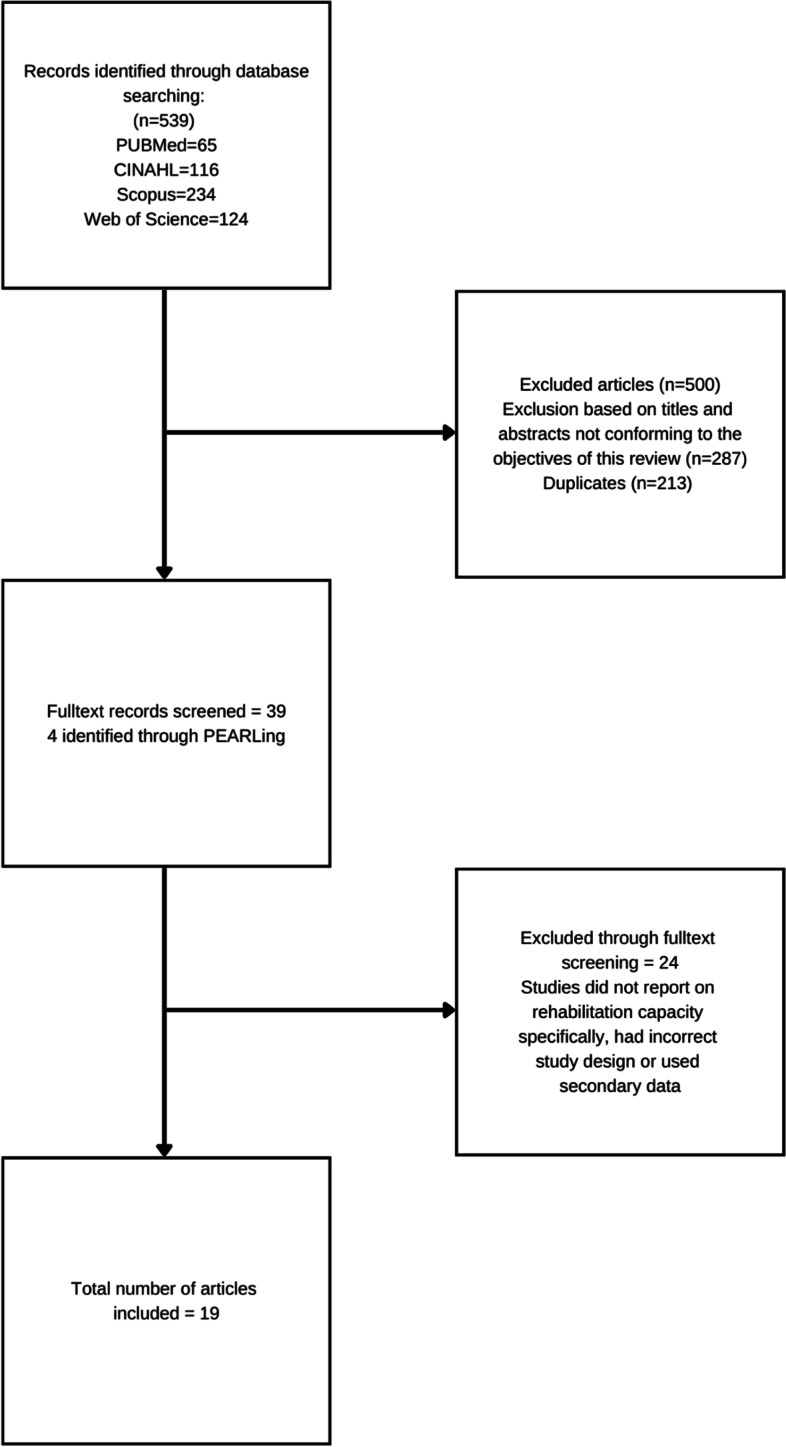
PRISMA flowchart showing the selection of studies for inclusion in the scoping review

The search produced 539 initial hits. Based on title and abstract screening, 500 records were excluded (213 duplicates and 287 that did not comply with the inclusion criteria). Full texts of the remaining 39 studies were screened and four further studies were identified via PEARLing. After full-text screening, a further 24 studies were excluded, and 19 studies remained for analysis (Fig. 1).

### Study characteristics

The included studies were published between 2005 and 2021. Eleven out of the 19 studies (57.9%) were published in the last five years. The total number of countries reported on the eligible studies, was 41 countries; with one study [4]  reporting on 35 HICs.

Among the four LMICs included, South Africa [17, 18] and Brazil [19, 20] are classified as upper-middle-income countries [21, 22], while Pakistan [23] and Bangladesh [24] are lower-middle-income countries [25, 26]. The United States of America (USA) was included in eight (42.1%) of the studies [4, 24, 27–32], Canada in five (26.3%) [4, 28, 33–35] and Australia in three (15.8%) [4, 36, 37].

One of the studies compared physiotherapy between Canada and the USA [28]. The data used in that study were sourced from one of the other studies reporting on Canada only [33]. The other two studies reporting on physiotherapy in Canada were both based in Saskatchewan and published in the same year, using the same data sources [34, 35]. One of these studies reported on the distribution of rural versus urban physiotherapists [34], and the other reported on primary health care (PHC) physiotherapists [35]. The fifth study reporting on Canada was the study comparing 35 HICs [4], which is also one of the studies that included the USA and Australia. Physiotherapy was the more common profession included in the studies (*n* = 14, 73.7%) [4, 19, 20, 23, 24, 27, 28, 32–38], and in nine (64.3%) of these studies, physiotherapy was the only profession reported on [20, 24, 27, 28, 33–36, 38].

Two (10.5%) studies [35, 37] described a specific level of care. One study (5.3%) described only the PHC [35] workforce, and one study looked at the workforce providing services to adults requiring inpatient general rehabilitation [37]. Another two (10.5%) studies [29, 32] described specific population groups. One (5.3%) study included the workforce licenced to issue hearing aids and considered the population ≥ 65 years of age with hearing loss [29]. Wilson et al. (2009) only reported on the ‘underserved area’ population group, classified according to workforce shortage areas [32]. Eighteen (94.7%) of the studies were database reviews and the remaining study was a cross-sectional survey [37] (Table 1).

**Table 1:** Summary of study characteristics

	**Country**	**Study design**	**Aim**
**Physiotherapy**			
Anderson, 2005	Australia ^a^	Database Review	To generate a profile of the physiotherapy profession in New South Wales
Landry, 2007	Canada ^a^	Database Review	To estimate HHR ratios across provincial jurisdictions by combined population data with lists of registered PTs
Landry, 2009	USA ^a^ & Canada ^a^	Database Review	To estimate PT HHR ratios across the USA to conduct a comparative analysis of USA and Canada
Zimbelman, 2010	USA ^a^	Database Review	To examine current and future PT job surplus/ shortage trends across the USA
Bath, 2015	Canada ^a^	Database Review	To compare demographics and clinical characteristics and map the distribution between PTs in rural & urban areas
Shah, 2015	Canada ^a^	Database Review	To investigate differences in geographic accessibility to community-based PTs and FPs
Jesus, 2016	USA ^a^, Singapore ^a^, Portugal ^a^, Bangladesh ^b^	Database Review	To examine the PT supply across 4 countries to reflect contextual factors likely to affect PT supply needs
Eighan, 2018	Ireland ^a^	Database Review	To estimate the supply of PTs in Ireland and profile PTs across acute and non-acute sectors and across public & private
Rodes, 2021	Brazil ^b^	Database Review	To describe secular trends of the PT workforce-to-population ratio among the public and private health care sectors and across care levels of the Unified Health System
**Occupational Therapy**			
Ned, 2020	South Africa ^b^	Database Review	To describe the demographic trends of occupational therapists from 2002 to 2018 in South Africa
**Audiology**			
Windmill, 2013	USA ^a^	Database Review	To apply the Physician Supply Model to audiology and determine supply of audios and if it will meet future demands
Planey, 2016	USA ^a^	Database Review	To assess the relationships between socio-demographic and structural factors and audiologist supply
Coco, 2018	USA ^a^	Database Review	To present results from a geographic analysis as part of teleaudiology planning assessment for Arizona
**Audiology & Speech Therapy**			
Pillay, 2020	SA ^b^	Database Review	To examine the demographic profile and supply of audiologists and speech therapists in South Africa
**Physiotherapy and Occupational Therapy**			
Rathore, 2011	Pakistan ^b^	Database Review	To present an overview of Physical Medicine and Rehabilitation in Pakistan, its origins, status and plans
Jesus, 2020	35 Countries ^a^	Database Review	To determine whether population-adjusted rates of rehabilitation needs are associated with the PT & OT supply across 35 HICs
**Physiotherapy, Occupational Therapy and Speech Therapy**			
Wilson, 2009	USA ^a^	Database Review	To assess the distribution of rehabilitation health professional shortages and the differences between metro/ non-metro counties
Barrett, 2015	Australia ^a^	Cross-sectional	To profile staffing levels of allied health professionals and support staff in Queensland Health inpatient services
Rodes, 2017	Brazil ^b^	Database Review	To estimate the distribution trend of rehabilitation HR in Brazilian HCN, especially for PHC of STs, OTs and PTs

*Legend*: *USA* United States of America, *PT* Physiotherapy, *HHR* Human health resources, *FP* Family physicians, *OT* Occupational therapy, *STs* Speech therapists, *HICs* High income countries, *LMICs* Low-to middle-income countries

^**a**^ HICs and ^b^ LMICs

### Rehabilitation data sources used in included studies

The data analysed by the included studies were retrieved from various publicly-accessible resources. More than half of the studies (*n* = 11, 57.9%)) used data from professional associations [4, 17, 18, 24, 28, 30, 31, 34–36, 38]. The professional associations were either regional (*n* = 3) [34–36], national (*n* = 7) [17, 18, 24, 28, 30, 31, 38] or international professional associations (*n* = 1) [3] and were either profession-specific (*n* = 9) [3, 24, 30, 31, 34–36, 38] or general health regulatory boards (*n* = 2) [17, 18]. Nine of the studies [17–20, 29, 32, 33, 36, 38] used general health databases and some used other government databases such as national census results or government bureau statistics. Only one [37] of the included studies conducted a cross-sectional study specifically aimed at describing rehabilitation workforce.

### Workforce descriptions 

#### Education

Two (10.5%) [34, 36] studies reported on the qualification or education of the rehabilitation workforce. One (5.3%) study reported on the number of physiotherapists with postgraduate degrees in New South Wales, Australia [36]. In 2001, 23% of physiotherapists had at least one postgraduate degree. This has increased significantly from 1975 when only 5.4% of physiotherapists had postgraduate qualifications. The second study [34] reported on three elements, namely the institution of qualification, the highest level of qualification, and whether the workforce had completed the Physiotherapy Competency Examination in Saskatchewan, Canada. Eighty-one percent of physiotherapists working in Saskatchewan qualified at the University of Saskatchewan, 13% qualified at another university in Canada and 6% qualified internationally. Most of the physiotherapists (69%) had a bachelor’s degree, 12% had a diploma or certificate and 19% had a postgraduate degree.

#### Age and gender

Six (31.6%) of the included studies reported on the age and/or gender of the rehabilitation workforce [17, 18, 30, 34, 36, 38]. Four of these studies examined both age and gender, one study reported on age only and the other study on gender only. Three of the studies that reported on the age of the workforce examined the number per age group [17, 18, 30]. One study reported the median age of physiotherapists working in Saskatchewan [34], and another study reported the modal age of physiotherapists per gender [36]. The remaining four studies that examined the gender of the rehabilitation workforce calculated the distribution between males and females [17, 18, 34, 38]. Table 2 shows that at least 70% of the rehabilitation workforce were female and that most of the population was < 40 years old. One study had an almost even distribution of audiologists between the age groups of 31–40, 41–50 and 51–60 years [30].

**Table 2:** Data on age and gender

	**Age**	**Gender**
**Physiotherapy**		
*Anderson, 2005*	Female Modal Age: 40–44 y Male Modal Age: 30–34 y	Female: 76.5% (*n* = 439.11) Male: 23.5% (*n* = 134.89)
*Bath, 2015*	Median age: ≤ 40 y	Female: 79% (*n* = 508) Male: 21% (*n* = 135)
*Eighan, 2018*	*Not reported*	Female: 74% (*n* = 1 940) Male: 26% (*n* = 677)
**Occupational Therapy**		
*Ned, 2020*	< 40y: 67.7% (*n* = 3019)	Female: 95% (n = 4193) Male: 5% (n = 267)
**Audiology**		
*Windmill, 2013*	Number in age range < 30 y: 11% (*n* = 1760) 31–40 y: 26% (*n* = 4160) 41–50 y: 25% (*n* = 4000) 51–60 y: 26% (*n* = 4160) > 60 y: 12% (*n* = 1920)	*Not reported*
**Audiology and Speech Therapy**		
*Pillay, 2020*	< 40y: 63.6% (*n* = 2078) > 50 y: 12.6% (*n* = 397)	Female: 94.6% (*n* = 3090) Male: 5.4% (*n* = 176)

#### Full-time versus part-time employment

Five (26.3%) studies [30, 34, 36–38] reported on either the distribution of the rehabilitation workforce between full-time and part-time or on the full-time equivalent (FTE) numbers. One study reported part-time as working less than 30 h a week [36]. Another study reported part-time status as working less than 20 h a week [30]. A third study reported part-time status as less than 37 h a week [38]. One study found that 39.4% of the physiotherapists, in New South Wales, worked part-time, of which 93.2% were female and 58.7% of the physiotherapists working full-time, 59% were female [36]. The second study reported that only 25% of audiologists in the USA worked part-time [30]. Audiologists that did not have a full-time clinical role were included in the 25%. One study reported 202 FTE, allied health staff, for 466 in-patient rehabilitation beds [37]. The fourth study reported that 17% of physiotherapists in Ireland worked part-time, with the FTE number at 2617 [38]. The fifth study reported that 25% of physiotherapists in Saskatchewan worked part-time [34].

#### Type of rehabilitation professions

Most studies reported on physiotherapy (*n* = 14; 73.7%), of which nine reported on physiotherapy only [20, 24, 27, 28, 33–36, 38]. Two studies compared physiotherapy to other professions. One of these studies compared physiotherapy with family physicians [34] and the second study compared physiotherapy with occupational therapy, as well as doctors and nurses, although the primary aim was to report on physiotherapy [24]. Three studies (15.8%) reported on audiology only [29–31]. One (5.3%) study reported on both audiology and speech therapy [18]. Six (31.6%) studies reported on occupational therapy [4, 17, 19, 23, 32, 37], with one study that reported on occupational therapy only [17]. Three studies reported on physiotherapy, occupational therapy and speech therapy [19, 32, 37]. One of these studies also reported on other allied health staff [37]. Another two studies reported on physiotherapy and occupational therapy [4, 23].

#### Rehabilitation workforce distribution between public versus private sector

The distribution of the rehabilitation workforce between the public and private sectors was reported in seven (36.8%) studies [17, 18, 20, 34–36, 38]. Five of these studies reported on physiotherapy [20, 34–36, 38], one on audiology and speech therapy [18], and one on occupational therapy [17]. Two of the four studies reported on the physiotherapy workforce in Saskatchewan, Canada [34, 35]. Both studies reported similar findings, namely that the gap between private and public are almost equally distributed, with public supply being just more than 50%. One study categorised the physiotherapy workforce into ‘own’ practice, ‘private’ and ‘public’ sectors [36]. For the purposes of this study, own practice and the private sector were combined. A study conducted in Ireland reported that they had physiotherapists working in either private or public sectors and 3% worked in both the private and public sectors [38]. The previous two studies had similar findings to the two conducted in Canada, with the difference of the distribution between the public and private sectors being small. The two studies conducted in South Africa had a very different distribution, where more than 70% were working in the private sector (see Table 3 for detail) [17, 18].

**Table 3:** Rehabilitation workforce distribution between public versus private sectors

	**Public**	**Private**
***Physiotherapy***		
Anderson, 2005^a^	41% (*n* = 1231)	59% (*n* = 1756)
Bath, 2015^a^	58% (*n* = 347)	42% (*n* = 250)
Shah, 2015^a^	53.9% (*n* = 301) Other: 6.6% (*n* = 37)	39.4% (*n* = 220)
Eighan, 2019^a^	53% (*n* = 1682) Public and Private: 107	43.6% (*n* = 1383)
Rodes, 2021	64.4% (*n* = 57,542)	35.6% (*n* = 31,089)
***Occupational therapy***		
Ned, 2020	25.2% (*n* = 1305)	74.8% (*n* = 3875)
***Speech therapy and audiology***		
Pillay, 2020	22% (*n* = 719)	78% (*n* = 2548)

^a^ High-income countries (HICs)

#### Rehabilitation workforce distribution between urban versus rural settings

Four (21.1%) of the 19 studies reported on the distribution of the rehabilitation workforce in urban versus rural areas (Table 4) [29, 32, 34, 36]. Two of these studies reported on physiotherapy only, where large differences were seen between the rural and urban distribution (Table 4) [34, 36]. One study reported on the distribution of physiotherapy, occupational therapy and speech therapy per 100 000 population [32]. The fourth study reported on the distribution of audiologists who provide hearing aids between rural and urban settings [29]. Table 4 shows that most of the rehabilitation workforce was located in an urban setting. In the study reporting on the physiotherapy, occupational therapy and speech therapy workforce, the difference was not as large as in the other three studies.

**Table 4:** Rehabilitation workforce distribution between urban and rural settings

	**Urban**	**Rural**
**Physiotherapy**		
Anderson, 2005^a^	80% (*n* = 5520)	20% (*n* = 1380)
Bath, 2015^a^	89% (*n* = 571)	11% (*n* = 72)
**Physiotherapy, occupational therapy, and speech therapy**		
Wilson 2009^a^	PT: 5.09/10 000 OT: 2.47/10 000 ST: 3.5/10 000	PT: 3.55 OT: 1.53 ST: 2.95
**Audiology**		
Coco, 2018^a^	94% (*n* = 829)	6% (*n* = 50)

*Legend*: *PT* Physiotherapy, *OT* Occupational therapy and ST-speech therapy

^a^ High-income countries (HICs)

#### Workforce distribution between levels of care

Five (26.3%) studies reported quantitative data: three studies on the distribution of the rehabilitation workforce per level of care [19, 20, 38] and the other two studies reported on data from a level of care [35, 37]. Both studies that hailed from Brazil [19, 20], reported on the level of care; however, the reports were on different levels of care. The study from 2017 [19] reported on PHC, specialised ambulatory care and hospital care. The study from 2021 [20] reported on primary, secondary and tertiary health care. The other study [38] reporting on physiotherapists per level of care, calculated the distribution between acute and non-acute care. There were 1774 physiotherapists working in non-acute care and 846 in acute care [38]. One study reported on the rehabilitation workforce capacity of general inpatient beds in Queensland, Australia (466 beds) [37]. One of the studies conducted on physiotherapists in Saskatchewan reported on the availability at PHC level [35].

### Rehabilitation workforce density/supply

#### Supply

Most (*n* = 14; 73.7%) of the studies [17, 18, 20, 23, 28–31, 33–38] reported the absolute total numbers of the rehabilitation workforce. Nine of these studies provided totals at the national level [17, 18, 20, 23, 28, 30, 31, 33, 38], and the other five at the regional level [29, 34–37]. Two of these five studies reported on totals not only for a specific region but also for a specific population [29, 37]. One study reported on the number of audiologists (*n* = 879) who serve the population who are most likely to have hearing impairments in Arizona, USA [29]. The other study in Queensland, Australia, reported on the FTE (*n* = 119) of general inpatient beds [37]. Two studies reported on the total physiotherapists (*n* = 643) in Saskatchewan, Canada [34, 35] and the third study reported on physiotherapists (*n* = 6900) in New South Wales, Australia [36]. Three of the studies reporting on national totals were conducted in Canada (*n* = 15 772), the USA (*n* = 167 810) and Ireland (*n* = 3172) for physiotherapy [28, 33, 38]. One of the studies reported on the physiotherapy and occupational therapy workforce in Pakistan (*n* = 1150) [23]. Two studies reported on the occupational therapy (*n* = 5180) and audiology and speech therapy (*n* = 3266) workforce in South Africa [17, 18]. See Table 5 for the supply of professions not included in the national ratios.

**Table 5:** Supply of professions (not included in national ratios)

Author, Year	Province or State	PT	OT	ST	AU
Anderson, 2005	NSW, Australia	*n* = 718	*Not reported*		
Wilson, 2009	USA ^a^	4.28/ 10 000	2.67/ 10 000	2.94/ 10 000	
Shah, 2015	Saskatchewan, Canada	4.6/ 10 000 ^b^ (*n* = 615)	*Not reported*		
Bath, 2015	Saskatchewan, Canada	5.97/ 10 000 (*n* = 617)	*Not reported*		
Barrett, 2015	Queensland, Australia ^c^	*44.5%* *(n* = *53)*	36.8% (*n* = 43.8)	18.82% (*n* = 22.4)	
Coco, 2018 ^d^	Arizona, USA	*Not reported*			4.25/ 10 000 ^e^ (*n* = 332)

*Legend*: *PT* Physiotherapy, *OT* Occupational therapy, *ST* Speech therapy, *AU* Audiology, *NSW* New South Wales and USA-United States of America

Ratios per 10 000 population; ^a^ Health shortage areas population, ^b^ PHC population, ^c^ General inpatient rehabilitation beds, ^d^ Audiologists registered to issue hearing aids, ^e^ Population ≥ 65 with hearing loss

#### Population-adjusted ratios

Thirteen (68.4%) of the studies reported on the population-adjusted ratios [4, 17–20, 24, 27, 28, 31–33, 35, 38].The population ratio varied from 1 000 per capita to 100 000. All four of the LMICs reported population-adjusted ratios of less than one per 10 000 capita [17–20, 24]. One of the studies compared the population-adjusted ratio of 35 HICs [4].

**Fig. 2:**
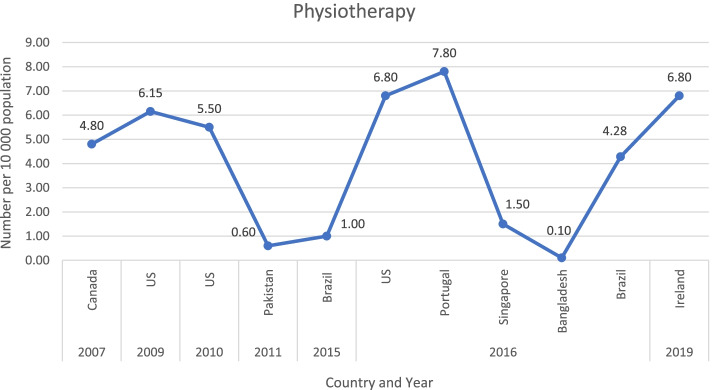
Comparison of population-adjusted ratios for physiotherapists. Legend: USA-United States of America

Figure 2 shows the ratio of physiotherapists per 10 000 population of those studies that reported ratios [19, 20, 24, 27, 28, 33, 38], as well as Rathore et al. (2011) that provided the total number [23, 39] (for the latter, the population ratio were calculated using population data from The World Bank (https://data.worldbank.org/indicator/SP.POP.TOTL)).

**Fig. 3:**
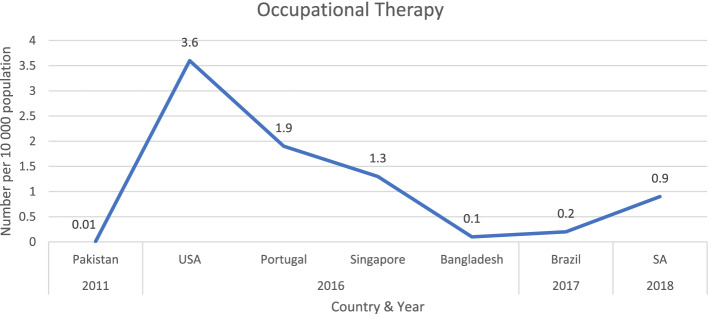
Comparison of population-adjusted ratios for occupational therapists. Legend: USA-United States of America and SA-South Africa

Figure 3 shows the ratios of occupational therapists per 10 000 population of those studies that reported ratios [17, 19, 24] and the ratio for Pakistan [23], for which the ratio was calculated as mentioned previously.

**Fig. 4:**
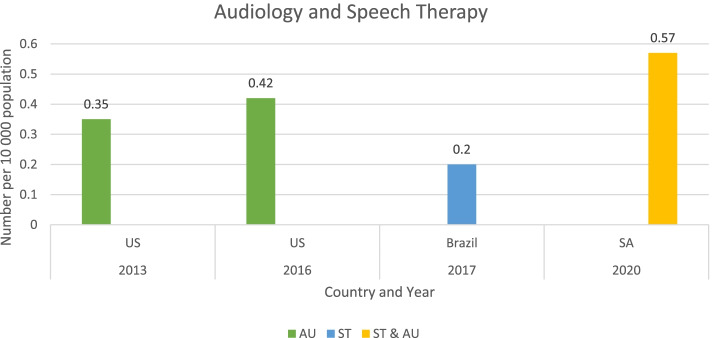
Comparison of population-adjusted ratios for speech therapy and audiology. Legend: USA-the United States of America, SA-South Africa, AU-audiology and ST-speech therapy

Figure 4 shows the ratios of audiologists and speech therapists reported in the study findings [18, 19, 30–32].

**Fig. 5:**
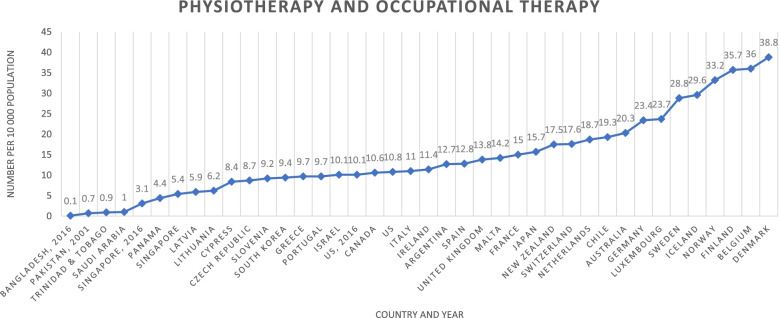
Physiotherapy and occupational therapy combined number per 10 000 population per country. Legend: USA-United States of America

Figure 5 shows the ratios of physiotherapy and occupational therapy combined per 10 000 population of the 35 HICs [4] and for the studies that provided data to calculate the combined ratios [19, 23, 24].

## Discussion

To our knowledge, this is the first scoping review that examined descriptors and indicators of the rehabilitation workforce capacity across core rehabilitation professions and on a global level. Although only four LMICs were included in the review, the main descriptors and indicators identified are arguably applicable across contexts and settings. Our findings indicated that the following descriptors and indicators were most commonly used to quantify the rehabilitation workforce across countries: profession type, density, supply, distribution between rural and urban regions, distribution between public and private sectors, and age and gender.

The most common way in which the rehabilitation workforce was indicated, related to profession type. Reporting the rehabilitation workforce by profession is important, as multidisciplinary rehabilitation is reported as being the most effective for delivering quality rehabilitation services [1, 3, 6]. In this review, physiotherapy was the dominant rehabilitation profession reported (reported in 74% [4, 19, 20, 23, 24, 27, 28, 32–38] of the included studies, while for example, only 25% reported on speech and language therapy [18, 19, 32, 37]). In LMICs where rehabilitation professionals are scarce, physiotherapy is often the only available rehabilitation profession [3, 5, 24], leading to sub-optimal services. Knowledge about the available rehabilitation professional types is thus also important for country-level planning of education and training opportunities for rehabilitation professionals to facilitate a multidisciplinary workforce [1]. Rathore et al. (2011) for example reported that there are only two occupational therapy training programmes in Pakistan, which contributed to the low number (*n* = 150) of occupational therapists [23]. Countries can therefore use information on the type of therapists to initiate new training programmes and devise interim strategies to ensure that comprehensive services are delivered.

A quarter (26%) of the included studies [17, 18, 30, 34, 36] reported on the age of the rehabilitation workforce. Many studies used publicly available datasets that did not specify data on age and gender. Anderson et al. (2005) commented that the modal age is a ‘statistic of great significance’ and that it can be a predictor of the profession’s longevity [36]. Ned et al. (2020) commented that a younger workforce can be due to poor absorption and retention rates in the public sector [17]. It may also be indicative of younger therapists working abroad. An ageing workforce implies that there is a risk that experience and skillsets are maintained to deliver quality services [17]. Therefore, it is important for countries to consider age when describing the rehabilitation workforce to ensure that human resource planning meets service demands.

In many countries, gender equity is an important goal and gender should thus be considered in the descriptors to track gender distribution. About 26% of the profession included gender in the description of the rehabilitation workforce [17, 18, 30, 36]. Since rehabilitation is still a predominantly female profession (this was the case for all studies that reported gender), it is advisable that future research and descriptions of the workforce consider gender as a descriptor. Although this trend is also notable in other healthcare professionals [40], countries should consider whether gender equity is an important consideration for the local context. In addition, operational factors such as the preference for part-time positions among females may have implications for service delivery. Therefore, local contextual factors play an important role in whether gender should be included as a descriptor of the rehabilitation workforce.

The distribution of the rehabilitation workforce between rural and urban settings was reported in about a fifth of included studies (four of the studies [29, 32, 34, 36]), while one other study only commented that there was a disparity between urban and rural settings [24]. Thus, there is sparse information on the rural or urban distribution of therapists. The studies that reported on the disparity between urban and rural rehabilitation workforce practices obtained their data from various sources, of which the credibility and comparison may be questionable. Two of the studies [34, 36] used the place of employment address requested from the professional body registration data. Other studies [29, 32] used data provided by universities or government registries. The lack of data regarding rural versus urban distribution is concerning, as it is generally known that there is a need for an increased workforce in rural areas [5, 35] and that such lacking a workforce leads to inequity in access. Bath et al. (2015) reported that rehabilitation workers in rural areas felt more isolated in terms of social and professional environments and were therefore unwilling to remain in the rural areas [34]. Without information on this vital indicator, policies and guidelines on workforce capacity cannot be changed to promote equity [3, 6]. Future studies should thus include information on the geographical (rural–urban) distribution of therapists.

Seven included studies (37%) [17, 18, 20, 34–36, 38] reported data on the distribution of the rehabilitation workforce between the public and private sectors. In the HICs [34–36, 38], there was not a large disparity between the number of therapists working in private versus public practice, but this trend is different in LMICs [17, 18] where most therapists work in private practice. In countries such as South Africa – where more than 80% of the population depends on public healthcare, which has less than 5% of the total resources for health – describing the public/private distribution of healthcare may be important. In other countries with universal healthcare coverage, this descriptor may be irrelevant. For countries where the private/public distribution of the workforce is relevant, it is important to ensure that this information is accurate and updated. National registries such as health registration bodies can be considered feasible data source options.

The included studies also reported on quantitative metrics to describe the rehabilitation workforce. These metrics included absolute values and population ratios. The population-adjusted ratio is the most general measure of workforce supply in a specific area, region or population [28]. Although population ratios provide an estimate of service coverage, additional factors – such as population needs [4] – should also be considered when describing the rehabilitation workforce using this metric. Thus, the interpretation of population ratios also requires an understanding of the local context. In addition, although these population ratios are commonly used, differences in scope of practice, type of professionals between countries and varying competencies across regions and countries may still bar accurate between-country comparisons [8]. Therefore, the between-country comparison still demands some degree of caution and trends may be more useful than absolute quantitative comparisons that consider contexts.

Our review shows that HICs could have a therapist density of up to 40 times higher than LMICs. The stark difference may be due to better financial resources and because HICs have integrated rehabilitation into their policies, which prioritise funding for rehabilitation. In many LMICs, rehabilitation remains under-recognised as an essential healthcare strategy. The triple burden of non-communicable and communicable conditions and trauma evident in LMICs, are all pressing needs, which often utilise most of the health resources and funding. Consequently, rehabilitation remains underfunded and not well integrated into service and human resource planning.

### Limitations

A single reviewer performed the searching and screening of titles and abstracts, which may have introduced bias. The review included only published primary studies; this may have led to the omission of unpublished information contained in the grey literature. Only studies published in English were included, which may have led to language bias and the potential exclusion of studies from some LMICs. The included studies were limited to studies reporting on rehabilitation workforce capacity, this excluded studies that were on rehabilitation workforce but did not include the capacity thereof – however, studies that did not report on capacity descriptors/metrics fell outside of the scope of this review. In line with scoping review methodology and the purpose of providing a comprehensive summary of the descriptors and metrics (rather than a critical synthesis), no critical appraisal of the included studies was performed [41].

### Recommendations

The study findings can contribute to further research on rehabilitation workforce capacity and/or inform a systematic review on rehabilitation workforce. The data and information on rehabilitation workforce capacity in LMICs are lacking, and there is therefore a need to increase reliable and accurate data in LMICs to inform policies and finances for rehabilitation workforce. Future studies should adopt a more in-depth search strategy, including grey literature, to include a broader range of data (descriptors and metrics) at regional and national levels (especially in LMICs where such data may not necessarily be published as research).

## Conclusion

This study aimed to scope the range of descriptors and metrics used to describe the rehabilitation workforce and to compare the workforce across countries that used similar descriptors. Despite the lack of a common definition or scope of the rehabilitation workforce capacity across countries, this review found the six most included descriptors in the eligible studies. The descriptors were profession type, density, supply, distribution between rural and urban regions, distribution between public and private sectors, age and gender. These descriptors enabled us to compare the capacity of the rehabilitation workforce across countries, keeping in mind the local context and economic status. These comparisons show that there is a large disparity in the rehabilitation workforce capacity between HICs and LMICs. With many LMICs already having poor access to rehabilitation services, this lack of workforce capacity means that access to rehabilitation services for the most vulnerable populations will be exacerbated. There is a need for reliable and appropriate data on rehabilitation workforce capacity, especially in LMICs, to strengthen rehabilitation into policies and promote the integration of rehabilitation in the local healthcare system.

## Supplementary Information

**Additional file 1. Addendum 1. **PRISMA Extension for Scoping Reviews checklist.

**Additional file 2: Addendum 2.** PubMed Search String.

**Additional file 3: Addendum 3.** Data extraction forms 1 and 2.

## Data Availability

All data generated and/or analysed during this study are included in this published article, except for the population data from relevant studies which were obtained from the World Bank repository, https://data.worldbank.org/indicator/SP.POP.TOTL.

## Abbreviations

LMICs: Low- to middle-income countries
HICs: High-income countries
WHO: World Health Organisation
USA: United States of America
PHC: Primary health care
FTE: Full-time equivalent
PT: Physiotherapy/physiotherapist
ST: Speech therapy/ therapist
OT: Occupational therapy/ therapist
AU: Audiologist/ Audiology
NSW: New South Wales
SA: South Africa
