# Supplementary material for: Rehabilitation workforce descriptors: a scoping review
Source: BMC Health Serv Res. 2022 Sep 17;22:1169. doi: 10.1186/s12913-022-08531-z (PMC9482289; doi:10.1186/s12913-022-08531-z)
Supplement: Supplementary file 2 — Additional file 2: Addendum 2. PubMed Search String. [file 12913_2022_8531_MOESM2_ESM.docx]

**Rehabilitation workforce descriptors: a scoping review**

Thandi Conradie^1^, Karina Berner^1^, Quinette Louw^1^

^1^Department of Health and Rehabilitation Sciences,

Faculty of Medicine and Health Sciences,

Stellenbosch University,

Cape Town, South Africa

**Corresponding author:** T Conradie, [thandic@sun.ac.za](mailto:thandic@sun.ac.za), +278422923723

## Addendum 2: PubMed Search String

| **Search number** | **Query** | **Results** |
| --- | --- | --- |
| 11 | (((Physical Therapy Specialty[Mesh] OR Occupational Therapy[Mesh] OR Speech Therapy[Mesh] OR Speech-Language Pathology[Mesh] OR Audiology[Mesh]) OR (Allied Health Occupations[Mesh] NOT "Medical Laboratory Science"[Mesh] NOT "Technology, Dental"[Mesh] NOT "Technology, Radiologic"[Mesh])) OR ((physiotherap*[Title/Abstract]) OR ("physical therap*"[Title/Abstract]) OR ("occupational therap*"[Title/Abstract]) OR ("speech language therap*"[Title/Abstract]) OR ("speech therap*"[Title/Abstract]) OR ("speech patholog*"[Title/Abstract]) OR ("speech language patholog*"[Title/Abstract]) OR (audiolog*[Title/Abstract]))) OR (rehabilitation[Title/Abstract]) | 258,717 |
| 10 | Demography[Mesh] OR "workforce demographics"[Title/Abstract] OR demographics[Title/Abstract] OR profile[Title/Abstract] OR diversity[Title/Abstract] OR data[Title/Abstract] OR trends[Title/Abstract] OR composition[Title/Abstract] OR characteristics[Title/Abstract] | 6,921,423 |
| 9 | number[Title/Abstract] OR "workforce supply"[Title/Abstract] OR capacity[Title/Abstract] OR supply[Title/Abstract] OR manpower[Title/Abstract] OR stock[Title/Abstract] | 2,650,452 |
| 8 | Workforce[Mesh] OR "Health Workforce"[Mesh] OR "rehabilitation workforce"[Title/Abstract] OR "human resources for health"[Title/Abstract] OR "allied health workforce"[Title/Abstract] OR "human resources"[Title/Abstract] | 84,763 |
| 7 | "Health Services Needs and Demand"[Mesh] OR "workforce needs"[Title/Abstract] OR needs[Title/Abstract] OR demand[Title/Abstract] OR "population needs"[Title/Abstract] | 515,372 |
| 6 | ((Physical Therapy Specialty[Mesh] OR Occupational Therapy[Mesh] OR Speech Therapy[Mesh] OR Speech-Language Pathology[Mesh] OR Audiology[Mesh]) OR (Allied Health Occupations[Mesh] NOT "Medical Laboratory Science"[Mesh] NOT "Technology, Dental"[Mesh] NOT "Technology, Radiologic"[Mesh])) OR ((physiotherap*[Title/Abstract]) OR ("physical therap*"[Title/Abstract]) OR ("occupational therap*"[Title/Abstract]) OR ("speech language therap*"[Title/Abstract]) OR ("speech therap*"[Title/Abstract]) OR ("speech patholog*"[Title/Abstract]) OR ("speech language patholog*"[Title/Abstract]) OR (audiolog*[Title/Abstract])) | 97,099 |
| 5 | (physiotherap*[Title/Abstract]) OR ("physical therap*"[Title/Abstract]) OR ("occupational therap*"[Title/Abstract]) OR ("speech language therap*"[Title/Abstract]) OR ("speech therap*"[Title/Abstract]) OR ("speech patholog*"[Title/Abstract]) OR ("speech language patholog*"[Title/Abstract]) OR (audiolog*[Title/Abstract]) | 84,718 |
| 3 | Physical Therapy Specialty[Mesh] OR Occupational Therapy[Mesh] OR Speech Therapy[Mesh] OR Speech-Language Pathology[Mesh] OR Audiology[Mesh] | 26,754 |
| 1 | Allied Health Occupations[Mesh] NOT "Medical Laboratory Science"[Mesh] NOT "Technology, Dental"[Mesh] NOT "Technology, Radiologic"[Mesh] | 21,392 |
