# Supplementary material for: Rehabilitation workforce descriptors: a scoping review
Source: BMC Health Serv Res. 2022 Sep 17;22:1169. doi: 10.1186/s12913-022-08531-z (PMC9482289; doi:10.1186/s12913-022-08531-z)
Supplement: Supplementary file 3 — Additional file 3: Addendum 3. Data extraction forms 1 and 2. [file 12913_2022_8531_MOESM3_ESM.docx]

**Rehabilitation workforce descriptors: a scoping review**

Thandi Conradie^1^, Karina Berner^1^, Quinette Louw^1^

^1^Department of Health and Rehabilitation Sciences,

Faculty of Medicine and Health Sciences,

Stellenbosch University,

Cape Town, South Africa

**Corresponding author:** T Conradie, [thandic@sun.ac.za](mailto:thandic@sun.ac.za), +278422923723

## Addendum 3: Data extraction form 1

| **Author, Year** | **Country** | **National or regional** | **World bank country economic classification** | **Study design** | **Data collection/ source** |
| --- | --- | --- | --- | --- | --- |
| **Physiotherapy** | | | | | |
| Anderson, 2005 | Australia | Regional | HIC | Database Review | Physiotherapy Labourforce Annual Survey Summary Reports, NSW Physiotherapists Registration Board, Department of Labour and Immigration, Australian Bureau of Statistics, Physiotherapists Registration Board, Australian Institute of Health & Welfare and Labour Force Surveys |
| Landry, 2007 | Canada | National | HIC | Database Review | Statistics Canada and Canadian Institute for Health Information |
| Shah, 2015 | Canada | Regional | HIC | Database Review | Saskatchewan College of Physical Therapists GIS Library Services, part of The Spatial Initiative at the University of Saskatchewan |
| Eighan, 2018 | Ireland | National | HIC | Database Review | Health Service Personnel Census and Irish Society of Chartered Physiotherapists Registered data. |
| Zimbelman, 2010 | US | National & regional | HIC | Database Review | U.S. Census Bureau, government public databases |
| Landry, 2009 | US & Canada | National & regional | HIC | Database Review | U.S. Census Bureau, government public databases, American Physical Therapy Association |
| Bath, 2015 | Canada | Regional | HIC | Database Review | • 2013 Saskatchewan College of Physical Therapists membership renewal • Saskatchewan Physiotherapy Association’s (SPA) 2012 membership list  • A list of physiotherapists who had served as clinical instructors |
| Jesus, 2016 | US,  Singapore,  Portugal,  Bangladesh | National | HIC HIC HIC LMIC | Database Review | • Local and international public domain data • Institutional reports (e.g., World Report on Disability) • Local and international websites (e.g., APTA, World Bank) • Authors’ qualitative accounts |
| **Audiology** | | | | | |
| Windmill, 2013 | US | National | HIC | Database Review | Membership survey of the members of the American Academy of Audiology |
| Planey, 2016 | US | National | HIC | Database Review | American Speech-Language Hearing Association membership dataset |
| Coco, 2018 | US | Regional | HIC | Database Review | US Census Arizona Department of Health Service and the US Veterans Administration |
| **Occupational therapy** | | | | | |
| Ned, 2020 | SA | National | LMIC | Database Review | HPCSA and PERSAL |
| **Speech therapy and audiology** | | | | | |
| Pillay, 2020 | SA | National | LMIC | Database Review | The data set was drawn from the Health Professions Council of South Africa (HPCSA) registers (for 2002–2017). PERSAL (available in SAHR 2017) |
| **Physiotherapy and occupational therapy** | | | | | |
| Jesus, 2020 | 35 Countries | National | HIC | Database Review | WCPT and WFOT |
| Rathore, 2011 | Pakistan | National | LMIC | Database Review | Federal Bureau of Statistics |
| **Physiotherapy, occupational therapy and speech therapy** | | | | | |
| Barrett, 2015 | Australia | Regional | HIC | Cross-sectional Survey | Survey with AH managers at facilities and Health Statistics Centre (Queensland Department of Health |
| Rodes, 2017 | Brazil | National & Regional | LMIC | Database Review | Brazilian National Register of Health Establishments |
| Wilson, 2009 | US | National | HIC | Database Review | Area Resource File (ARF), Equal Employment Opportunity, US Census for 2000, Health Resources and Services Administration and US Office of Management and Budget |

## Addendum 3: Data extraction form 2

| **Author, Year** | **Education** | **Demographics** | **Rural/Urban** | **Private/Public** | **Levels of care** | **Indicators** |
| --- | --- | --- | --- | --- | --- | --- |
| **Physiotherapy** | | | | | | |
| Anderson, 2005 | PG Degrees: 23.1% | Age & Gender - F: 40-44 y  M: 30-34 y | Urban: 80 % (Rural: 20%) | Private: 1756 Public: 1231 |  | Absolute Total: 574 |
| Landry, 2007 |  |  |  | |  | Ratio: 4.8/ 10 000 population |
| Landry, 2009 |  |  |  | |  | Total: US: 167 810 Canada: 15 772 |
|  |  |  |  |  |  | Ratio: US: 6.2/ 10 000  Canada: 4.8/ 10 000 |
| Zimbelman, 2010 |  |  |  | |  | Ratio: 5.5/ 10 000 population |
| Bath, 2015 | University of Saskatchewan: 521 (81.0)  Other Canadian: 84 (13.1)  International institute: 38 (5.9) | Gender: F- 508 (79.0)  M- 135 (21.0)  Median age: -≤40  Full time: 471 (74.9)  Part time: 156 (25.1) | Rural:72 (11.2%)  Urban: 571 | Public: 347 (58.1)  Private: 250 (41.9) |  | Total: 643 |
|  | Diploma/ certificate: 78 (12.1%)  Bachelor’s degree: 444 (69.1%)  Master’/doctoral degree: 121 (18.8%) |  |  |  |  |  |
| Shah, 2015 |  |  |  | Public: 301 Private: 220 Other: 37 | PHC | Total: 558 |
| Jesus, 2016 |  |  |  |  |  | Ratio- Portugal: 7.8/ 10 000   US: 6.5/ 10 000  Singapore: 1.5/ 10 000  Bangladesh: ≥0.1/10 000 |
| Eighan, 2018 |  | Female: 74% |  | Acute- Public: 776  Private: 118  Non-acute- Public: 871.7  Private: 899.4 | Non-acute: 2278 Acute: 894 | Total: 3172 WTE: 2617 |
| **Audiology** | | | | | | |
| Coco, 2018 |  |  | Urban: 94% (829) Rural: 6% |  |  | Total: 332  Average per county: 6,049:1 (SD: 5,119) (population to provider ratio) |
| Windmill, 2013 |  | Age (FTE): <30 - 1232  31-40- 2912  41-50- 2800  51-60 - 2912  >60 - 1344 |  |  |  | Total: 16 000 Clinical: 12 800 FTE: 11 200 |
| Planey, 2016 |  |  |  |  |  | State: 2.1 - 7.62/ 100 000 Total: 13 479/ 100 000 Range per county: 0-197/ 100 000 Mean per county: ± 4/ 100 000 |
| **Occupational therapy** | | | | | | |
| Ned, 2020 |  | Age - <40y: 67.7% (n=3019)  Female: 95% (n = 4193)  Male: 5% (n = 267) |  | Public: 25.2% (n=1305)  Private: 74.8% (n=3875) |  | Total: 5147  Total ratio: 0.91/ 10 000 |
| **Speech therapy and audiology** | | | | | | |
| Pillay, 2020 |  | STA: 46.8%; ST: 33.3%; AU: 18.9% |  | Public: 22% Private: 78% |  | Total: 3266  Total ratio: 0.57/ 10 000 |
|  |  | Female: 94.6%; Male: 5.4% |  |  |  |  |
|  |  | • 25–29-year: 27.6% (902) • 30-35 years: 20.4% (665) • <40 years: 63.6% (2078) • >50 years: 12.6% (397) |  |  |  |  |
| **Physiotherapy and occupational therapy** | | | | | | |
| Rathore, 2011 |  | PT: 1000; OT: 150 |  |  | | Total: 1150 |
| Jesus, 2020 |  |  |  |  |  | Ratio: Mean- 15.7/ 10 000  SD- 10/ 10 000  Range- 0.9-38.7/ 10 000 |
| **Physiotherapy, occupational therapy and speech therapy** | | | | | | |
| Barrett, 2015 |  | PT: 53; OT: 43.8; ST: 22.4 |  |  | Inpatient rehabilitation beds (n=466) | FTE = 119 |
| Rodes, 2017 |  |  |  |  | National • PHC - PT: <0.1/ 1000  OT: <0.1/1000  ST: <0.1/1000 • SAC - PT: 0.2/1000  OT: <0.1/1000  ST: 0.1/1000 • HC: PT: 0.12/1000  OT: <0.1/1000  ST: <0.1/1000 | Ratio per profession and level of care at national, state and city |
| Wilson, 2009 |  |  | Ratio  Urban PT: 50.9/100 000 OT: 24.7/100 000 ST: 35.0/100 000  Rural PT: 35.5/100 000 OT: 24.7/100 000 ST: 29.5/100 000 |  |  |  |
